# Supplementary material for: Enhancing Phosphorus and Nitrogen Uptake in Maize Crops with Food Industry Biosolids and Azotobacter nigricans
Source: Plants (Basel). 2023 Aug 25;12(17):3052. doi: 10.3390/plants12173052 (PMC10489705; doi:10.3390/plants12173052)
Supplement: Supplementary file 1 [file plants-12-03052-s001.zip › plants-2440271-supplementary.pdf]

Supplementary Figures

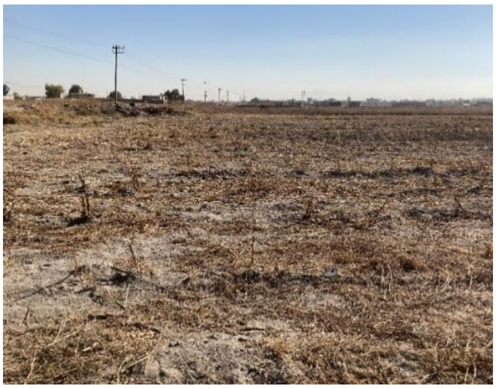

Figure S1. Parcel area of the Ex-Hacienda de Santa Inés, selected for the present study.

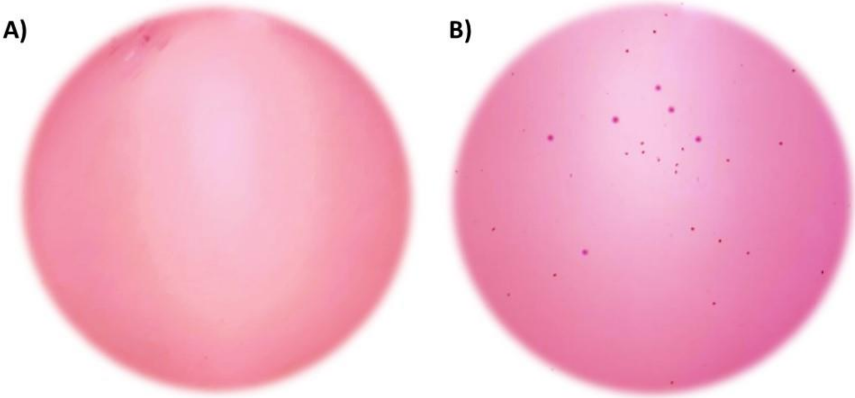

Figure S2. ABRV medium for count of total coliforms in biosolids a) Blank and b) Biological, dilution $1{\times}10^{-3}$ CFU mL^{-1}.

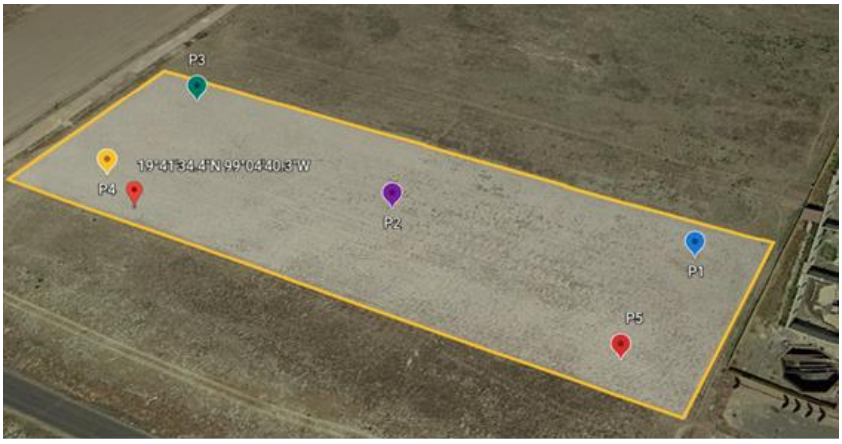

Figure S3. Plot of 500 m x 250 m in the Ex-Hacienda Santa Inés, Nextlalpan, Edo. Mex. (19°41'32.4"N 99°04'39.4"W), selected for agricultural soil sampling.

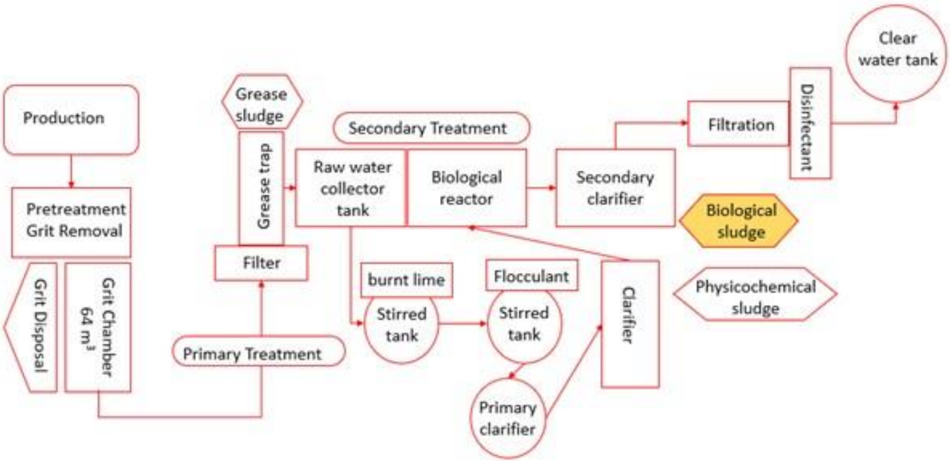

Figure S4. Wastewater treatment plant for the food industry of Grupo Herdez Mexico.

Supplemenytary Tables

Table S1. Three-way MANOVA. *Zea mays* seedlings grown with soil, biosolid 25% (v/v) and Long Ashton, with and without *Azotobacter nigricans* (1×10^8 CFU mL^{-1}).

Contrastes multivariados[d]

Efecto		Valor	F	Gl de la hipótesis	Gl del error	Sig.	Eta al cuadrado parcial	Parámetro de no centralidad Parámetro	Potencia observada[b]
Intersección	Traza de Pillai	1.000	30297.450[a]	4.000	21.000	.000	1.000	121189.801	1.000
	Lambda de Wilks	.000	30297.450[a]	4.000	21.000	.000	1.000	121189.801	1.000
	Traza de Hotelling	5770.943	30297.450[a]	4.000	21.000	.000	1.000	121189.801	1.000
	Raíz mayor de Roy	5770.943	30297.450[a]	4.000	21.000	.000	1.000	121189.801	1.000
Time	Traza de Pillai	1.984	672.031	8.000	44.000	.000	.992	5376.248	1.000
	Lambda de Wilks	.000	1316.319[a]	8.000	42.000	.000	.996	10530.552	1.000
	Traza de Hotelling	1026.785	2566.962	8.000	40.000	.000	.998	20535.698	1.000
	Raíz mayor de Roy	961.982	5290.903[c]	4.000	22.000	.000	.999	21163.612	1.000
Solid_matrix	Traza de Pillai	2.579	35.240	12.000	69.000	.000	.860	422.878	1.000
	Lambda de Wilks	.000	199.101	12.000	55.852	.000	.964	1509.051	1.000
	Traza de Hotelling	547.969	898.061	12.000	59.000	.000	.995	10776.729	1.000
	Raíz mayor de Roy	532.702	3063.038[c]	4.000	23.000	.000	.998	12252.154	1.000
Inoculum	Traza de Pillai	.985	353.946[a]	4.000	21.000	.000	.985	1415.783	1.000
	Lambda de Wilks	.015	353.946[a]	4.000	21.000	.000	.985	1415.783	1.000
	Traza de Hotelling	67.418	353.946[a]	4.000	21.000	.000	.985	1415.783	1.000
	Raíz mayor de Roy	67.418	353.946[a]	4.000	21.000	.000	.985	1415.783	1.000
Time * Solid_matrix	Traza de Pillai	2.956	11.329	24.000	96.000	.000	.739	271.895	1.000
	Lambda de Wilks	.000	48.592	24.000	74.470	.000	.914	791.414	1.000
	Traza de Hotelling	181.773	147.691	24.000	78.000	.000	.978	3544.580	1.000
	Raíz mayor de Roy	159.720	638.880[c]	6.000	24.000	.000	.994	3833.278	1.000
Time * Inoculum	Traza de Pillai	1.177	7.864	8.000	44.000	.000	.588	62.912	1.000
	Lambda de Wilks	.034	23.354[a]	8.000	42.000	.000	.816	186.829	1.000
	Traza de Hotelling	22.433	56.083	8.000	40.000	.000	.918	448.667	1.000
	Raíz mayor de Roy	22.151	121.831[c]	4.000	22.000	.000	.957	487.326	1.000
Solid_matrix * Inoculum	Traza de Pillai	1.945	10.594	12.000	69.000	.000	.648	127.125	1.000
	Lambda de Wilks	.015	17.943	12.000	55.852	.000	.752	169.162	1.000
	Traza de Hotelling	17.016	27.887	12.000	59.000	.000	.850	334.643	1.000
	Raíz mayor de Roy	14.944	85.931[c]	4.000	23.000	.000	.937	343.722	1.000
Time * Solid_matrix * Inoculum	Traza de Pillai	2.404	6.024	24.000	96.000	.000	.601	144.574	1.000
	Lambda de Wilks	.003	13.929	24.000	74.470	.000	.774	254.328	1.000
	Traza de Hotelling	30.511	24.790	24.000	78.000	.000	.884	594.961	1.000
	Raíz mayor de Roy	22.394	89.575[c]	6.000	24.000	.000	.957	537.448	1.000

a. Estadístico exacto

b. Calculado con alfa = .05

c. El estadístico es un límite superior para la F el cual ofrece un límite inferior para el nivel de significación.

d. Diseño: Intersección + Time + Solid_matrix + Inoculum + Time * Solid_matrix + Time * Inoculum + Solid_matrix * Inoculum + Time * Solid_matrix * Inoculum

Pruebas de los efectos inter-sujetos

Origen	Variable dependiente	Suma de cuadrados tipo III	gl	Media cuadrática	F	Sig.	Eta al cuadrado parcial	Parámetro de no centralidad Parámetro	Potencia observada[b]
Modelo corregido	Stem tickness (cm)	.232[a]	23	.010	39.389	.000	.974	905.943	1.000
	No. roots	174.979[c]	23	7.608	8.115	.000	.886	186.644	1.000
	Plant length (cm)	5851.872[d]	23	254.429	886.172	.000	.999	20381.957	1.000
	Root length (cm)	3167.150[e]	23	137.702	987.998	.000	.999	22723.945	1.000
Intersección	Stem tickness (cm)	7.434	1	7.434	29010.740	.000	.999	29010.740	1.000
	No. roots	2867.521	1	2867.521	3058.689	.000	.992	3058.689	1.000
	Plant length (cm)	22691.778	1	22691.778	79035.020	.000	1.000	79035.020	1.000
	Root length (cm)	9371.635	1	9371.635	67240.432	.000	1.000	67240.432	1.000
Time	Stem tickness (cm)	.121	2	.061	236.325	.000	.952	472.650	1.000
	No. roots	1.167	2	.583	.622	.545	.049	1.244	.142
	Plant length (cm)	5525.718	2	2762.859	9622.983	.000	.999	19245.967	1.000
	Root length (cm)	734.338	2	367.169	2634.396	.000	.995	5268.792	1.000
Solid_matrix	Stem tickness (cm)	.012	3	.004	15.098	.000	.654	45.293	1.000
	No. roots	3.562	3	1.187	1.267	.308	.137	3.800	.295
	Plant length (cm)	78.362	3	26.121	90.978	.000	.919	272.935	1.000
	Root length (cm)	1643.201	3	547.734	3929.927	.000	.998	11789.780	1.000
Inoculum	Stem tickness (cm)	.055	1	.055	216.008	.000	.900	216.008	1.000
	No. roots	99.187	1	99.187	105.800	.000	.815	105.800	1.000
	Plant length (cm)	95.739	1	95.739	333.458	.000	.933	333.458	1.000
	Root length (cm)	150.167	1	150.167	1077.430	.000	.978	1077.430	1.000
Time * Solid_matrix	Stem tickness (cm)	.023	6	.004	14.797	.000	.787	88.780	1.000
	No. roots	30.500	6	5.083	5.422	.001	.575	32.533	.980
	Plant length (cm)	95.002	6	15.834	55.149	.000	.932	330.891	1.000
	Root length (cm)	493.504	6	82.251	590.139	.000	.993	3540.834	1.000
Time * Inoculum	Stem tickness (cm)	.012	2	.006	22.472	.000	.652	44.943	1.000
	No. roots	6.000	2	3.000	3.200	.059	.211	6.400	.556
	Plant length (cm)	.100	2	.050	.174	.841	.014	.348	.074
	Root length (cm)	48.604	2	24.302	174.363	.000	.936	348.726	1.000
Solid_matrix * Inoculum	Stem tickness (cm)	.005	3	.002	6.967	.002	.466	20.902	.956
	No. roots	6.562	3	2.187	2.333	.099	.226	7.000	.515
	Plant length (cm)	14.669	3	4.890	17.031	.000	.680	51.093	1.000
	Root length (cm)	30.949	3	10.316	74.018	.000	.902	222.055	1.000
Time * Solid_matrix * Inoculum	Stem tickness (cm)	.004	6	.001	2.894	.029	.420	17.366	.796
	No. roots	28.000	6	4.667	4.978	.002	.554	29.867	.969
	Plant length (cm)	42.281	6	7.047	24.544	.000	.860	147.265	1.000
	Root length (cm)	66.388	6	11.065	79.388	.000	.952	476.326	1.000
Error	Stem tickness (cm)	.006	24	.000					
	No. roots	22.500	24	.938					
	Plant length (cm)	6.891	24	.287					
	Root length (cm)	3.345	24	.139					
Total	Stem tickness (cm)	7.672	48						
	No. roots	3065.000	48						
	Plant length (cm)	28550.540	48						
	Root length (cm)	12542.130	48						
Total corregida	Stem tickness (cm)	.238	47						
	No. roots	197.479	47						
	Plant length (cm)	5858.763	47						
	Root length (cm)	3170.495	47						

a. R cuadrado = .974 (R cuadrado corregida = .949)
b. Calculado con alfa = .05
c. R cuadrado = .886 (R cuadrado corregida = .777)
d. R cuadrado = .999 (R cuadrado corregida = .998)
e. R cuadrado = .999 (R cuadrado corregida = .998)

Table S2. Three-way MANOVA. Multivariate contrasts. *Zea mays* seedlings grown with soil, biosolid 25 (v/v) and Long Ashton, with and without *Azotobacter nigricans* (1×10^8 CFU mL^{-1}).

Contrastes multivariados[c]

Efecto		Valor	F	Gl de la hipótesis	Gl del error	Sig.
Intersección	Traza de Pillai	1.000	30297.450[a]	4.000	21.000	.000
	Lambda de Wilks	.000	30297.450[a]	4.000	21.000	.000
	Traza de Hotelling	5770.943	30297.450[a]	4.000	21.000	.000
	Raíz mayor de Roy	5770.943	30297.450[a]	4.000	21.000	.000
Time	Traza de Pillai	1.984	672.031	8.000	44.000	.000
	Lambda de Wilks	.000	1316.319[a]	8.000	42.000	.000
	Traza de Hotelling	1026.785	2566.962	8.000	40.000	.000
	Raíz mayor de Roy	961.982	5290.903[b]	4.000	22.000	.000
Solid_matrix	Traza de Pillai	2.579	35.240	12.000	69.000	.000
	Lambda de Wilks	.000	199.101	12.000	55.852	.000
	Traza de Hotelling	547.969	898.061	12.000	59.000	.000
	Raíz mayor de Roy	532.702	3063.038[b]	4.000	23.000	.000
inoculum	Traza de Pillai	.985	353.946[a]	4.000	21.000	.000
	Lambda de Wilks	.015	353.946[a]	4.000	21.000	.000
	Traza de Hotelling	67.418	353.946[a]	4.000	21.000	.000
	Raíz mayor de Roy	67.418	353.946[a]	4.000	21.000	.000
Time * Solid_matrix	Traza de Pillai	2.956	11.329	24.000	96.000	.000
	Lambda de Wilks	.000	48.592	24.000	74.470	.000
	Traza de Hotelling	181.773	147.691	24.000	78.000	.000
	Raíz mayor de Roy	159.720	638.880[b]	6.000	24.000	.000
Time * inoculum	Traza de Pillai	1.177	7.864	8.000	44.000	.000
	Lambda de Wilks	.034	23.354[a]	8.000	42.000	.000
	Traza de Hotelling	22.433	56.083	8.000	40.000	.000
	Raíz mayor de Roy	22.151	121.831[b]	4.000	22.000	.000
Solid_matrix * inoculum	Traza de Pillai	1.945	10.594	12.000	69.000	.000
	Lambda de Wilks	.015	17.943	12.000	55.852	.000
	Traza de Hotelling	17.016	27.887	12.000	59.000	.000
	Raíz mayor de Roy	14.944	85.931[b]	4.000	23.000	.000
Time * Solid_matrix * inoculum	Traza de Pillai	2.404	6.024	24.000	96.000	.000
	Lambda de Wilks	.003	13.929	24.000	74.470	.000
	Traza de Hotelling	30.511	24.790	24.000	78.000	.000
	Raíz mayor de Roy	22.394	89.575[b]	6.000	24.000	.000

a. Estadístico exacto

b. El estadístico es un límite superior para la F el cual ofrece un límite inferior para el nivel de significación.

c. Diseño: Intersección + Time + Solid_matrix + inoculum + Time * Solid_matrix + Time * inoculum + Solid_matrix * inoculum + Time * Solid_matrix * inoculum

Table S3. Three-way MANOVA. Comparison of means.

Comparaciones múltiples

Variable dependiente		(I)Solid matrix	(J)Solid matrix	Diferencia de medias (I-J)	Error típ.	Sig.	Intervalo de confianza 95%	
							Límite inferior	Límite superior
Stem thickness (cm)	DHS de Tukey	0.25Bios	Biosolid	.0417*	.00654	.000	.0236	.0597
			LA	.0317*	.00654	.000	.0136	.0497
			Soil	.0192*	.00654	.034	.0011	.0372
		Biosolid	0.25Bios	-.0417*	.00654	.000	-.0597	-.0236
			LA	-.0100	.00654	.436	-.0280	.0080
			Soil	-.0225*	.00654	.011	-.0405	-.0045
		LA	0.25Bios	-.0317*	.00654	.000	-.0497	-.0136
			Biosolid	.0100	.00654	.436	-.0080	.0280
			Soil	-.0125	.00654	.249	-.0305	.0055
		Soil	0.25Bios	-.0192*	.00654	.034	-.0372	-.0011
			Biosolid	.0225*	.00654	.011	.0045	.0405
			LA	.0125	.00654	.249	-.0055	.0305
	Bonferroni	0.25Bios	Biosolid	.0417*	.00654	.000	.0229	.0605
			LA	.0317*	.00654	.000	.0129	.0505
			Soil	.0192*	.00654	.044	.0004	.0380
		Biosolid	0.25Bios	-.0417*	.00654	.000	-.0605	-.0229
			LA	-.0100	.00654	.834	-.0288	.0088
			Soil	-.0225*	.00654	.013	-.0413	-.0037
		LA	0.25Bios	-.0317*	.00654	.000	-.0505	-.0129
			Biosolid	.0100	.00654	.834	-.0088	.0288
			Soil	-.0125	.00654	.407	-.0313	.0063
		Soil	0.25Bios	-.0192*	.00654	.044	-.0380	-.0004
			Biosolid	.0225*	.00654	.013	.0037	.0413
			LA	.0125	.00654	.407	-.0063	.0313
Root number	DHS de Tukey	0.25Bios	Biosolid	.50	.395	.593	-.59	1.59
			LA	.50	.395	.593	-.59	1.59
			Soil	.75	.395	.256	-.34	1.84
		Biosolid	0.25Bios	-.50	.395	.593	-1.59	.59
			LA	.00	.395	1.000	-1.09	1.09
			Soil	.25	.395	.921	-.84	1.34
		LA	0.25Bios	-.50	.395	.593	-1.59	.59
			Biosolid	.00	.395	1.000	-1.09	1.09
			Soil	.25	.395	.921	-.84	1.34
		Soil	0.25Bios	-.75	.395	.256	-1.84	.34
			Biosolid	-.25	.395	.921	-1.34	.84
			LA	-.25	.395	.921	-1.34	.84
	Bonferroni	0.25Bios	Biosolid	.50	.395	1.000	-.64	1.64
			LA	.50	.395	1.000	-.64	1.64
			Soil	.75	.395	.419	-.39	1.89
		Biosolid	0.25Bios	-.50	.395	1.000	-1.64	.64
			LA	.00	.395	1.000	-1.14	1.14
			Soil	.25	.395	1.000	-.89	1.39
		LA	0.25Bios	-.50	.395	1.000	-1.64	.64
			Biosolid	.00	.395	1.000	-1.14	1.14
			Soil	.25	.395	1.000	-.89	1.39
		Soil	0.25Bios	-.75	.395	.419	-1.89	.39
			Biosolid	-.25	.395	1.000	-1.39	.89
			LA	-.25	.395	1.000	-1.39	.89
Plant length (cm)	DHS de Tukey	0.25Bios	Biosolid	1.8392*	.21875	.000	1.2357	2.4426
			LA	-1.4083*	.21875	.000	-2.0118	-.8049
			Soil	-1.1350*	.21875	.000	-1.7384	-.5316

		Biosolid	0.25Bios	-1.8392*	.21875	.000	-2.4426	-1.2357
			LA	-3.2475*	.21875	.000	-3.8509	-2.6441
			Soil	-2.9742*	.21875	.000	-3.5776	-2.3707
		LA	0.25Bios	1.4083*	.21875	.000	.8049	2.0118
			Biosolid	3.2475*	.21875	.000	2.6441	3.8509
			Soil	.2733	.21875	.603	-.3301	.8768
		Soil	0.25Bios	1.1350*	.21875	.000	.5316	1.7384
			Biosolid	2.9742*	.21875	.000	2.3707	3.5776
			LA	-.2733	.21875	.603	-.8768	.3301
	Bonferroni	0.25Bios	Biosolid	1.8392*	.21875	.000	1.2102	2.4681
			LA	-1.4083*	.21875	.000	-2.0373	-.7794
			Soil	-1.1350*	.21875	.000	-1.7639	-.5061
		Biosolid	0.25Bios	-1.8392*	.21875	.000	-2.4681	-1.2102
			LA	-3.2475*	.21875	.000	-3.8764	-2.6186
			Soil	-2.9742*	.21875	.000	-3.6031	-2.3452
		LA	0.25Bios	1.4083*	.21875	.000	.7794	2.0373
			Biosolid	3.2475*	.21875	.000	2.6186	3.8764
			Soil	.2733	.21875	1.000	-.3556	.9023
		Soil	0.25Bios	1.1350*	.21875	.000	.5061	1.7639
			Biosolid	2.9742*	.21875	.000	2.3452	3.6031
			LA	-.2733	.21875	1.000	-.9023	.3556
Root Length (cm)	DHS de Tukey	0.25Bios	Biosolid	2.9083*	.15241	.000	2.4879	3.3288
			LA	5.5250*	.15241	.000	5.1046	5.9454
			Soil	-9.9250*	.15241	.000	-10.3454	-9.5046
		Biosolid	0.25Bios	-2.9083*	.15241	.000	-3.3288	-2.4879
			LA	2.6167*	.15241	.000	2.1962	3.0371
			Soil	-12.8333*	.15241	.000	-13.2538	-12.4129
		LA	0.25Bios	-5.5250*	.15241	.000	-5.9454	-5.1046
			Biosolid	-2.6167*	.15241	.000	-3.0371	-2.1962
			Soil	-15.4500*	.15241	.000	-15.8704	-15.0296
		Soil	0.25Bios	9.9250*	.15241	.000	9.5046	10.3454
			Biosolid	12.8333*	.15241	.000	12.4129	13.2538
			LA	15.4500*	.15241	.000	15.0296	15.8704
	Bonferroni	0.25Bios	Biosolid	2.9083*	.15241	.000	2.4701	3.3465
			LA	5.5250*	.15241	.000	5.0868	5.9632
			Soil	-9.9250*	.15241	.000	-10.3632	-9.4868
		Biosolid	0.25Bios	-2.9083*	.15241	.000	-3.3465	-2.4701
			LA	2.6167*	.15241	.000	2.1785	3.0549
			Soil	-12.8333*	.15241	.000	-13.2715	-12.3951
		LA	0.25Bios	-5.5250*	.15241	.000	-5.9632	-5.0868
			Biosolid	-2.6167*	.15241	.000	-3.0549	-2.1785
			Soil	-15.4500*	.15241	.000	-15.8882	-15.0118
		Soil	0.25Bios	9.9250*	.15241	.000	9.4868	10.3632
			Biosolid	12.8333*	.15241	.000	12.3951	13.2715
			LA	15.4500*	.15241	.000	15.0118	15.8882

Basadas en las medias observadas.
El término de error es la media cuadrática(Error) = .139.
*. La diferencia de medias es significativa al nivel .05.
